# PD-1 inhibitor plus oncolytic vaccinia virus is a safe and effective treatment option for metastatic renal cell carcinoma

**DOI:** 10.1186/s12935-024-03238-z

**Published:** 2024-01-30

**Authors:** Jee Soo Park, Myung Eun Lee, Jongchan Kim, Keunhee Oh, Namhee Lee, Minsun Jung, Won Sik Jang, Won Sik Ham

**Affiliations:** 1 Department of Urology and Urological Science Institute, Yonsei University College of Medicine, Seoul, Republic of Korea; 2 Department of Urology, Yongin Severance Hospital, Yonsei University Health System, Yongin, Republic of Korea; 3 Research Center, SillaJen, Inc., Yongin-si, Gyeonggi-do Republic of Korea; 4 Department of Pathology, Yonsei University College of Medicine, Seoul, Republic of Korea

**Keywords:** Renal cell carcinoma, Oncolytic viruses, Immunotherapy, Immune-related adverse events, Hepatitis

## Abstract

**Background:**

Although a combination of immune checkpoint inhibitors (ICIs) is recommended as the first line treatment option for metastatic renal cell carcinoma (mRCC), several immune-related adverse events (irAEs) occur, especially hepatitis. We explored the therapeutic benefits and safety profile of combining oncolytic vaccinia virus, JX-594, with a programmed cell death protein-1 (PD-1) inhibitor.

**Methods:**

We used early-stage and advanced-stage orthotopic murine mRCC models developed by our group. PD-1 inhibitor monotherapy or a PD-1 inhibitor combined with either JX-594 or a cytotoxic T-lymphocyte-associated antigen 4 (CTLA-4) inhibitor were systemically injected through the peritoneum. An immunofluorescence analysis was performed to analyze the tumor immune microenvironment (TIME). irAEs were assessed in terms of hepatitis.

**Results:**

In the early-stage mRCC model mice, the combination of JX-594 and a PD-1 inhibitor significantly decreased the primary tumor size and number of lung nodules, compared with the ICI combination, but the JX-594 and PD-1 inhibitor combination and ICI combination did not differ significantly in the advanced-stage mRCC model mice. The JX-594 and PD-1 inhibitor combination induced tumor-suppressing TIME changes in both the early- and advanced-stage mRCC models. Furthermore, mice treated with the ICI combination had significantly greater hepatic injuries than those treated with the JX-594 and PD-1 inhibitor combination which was evaluated in early-stage mRCC model.

**Conclusions:**

The JX-594 and PD-1 inhibitor combination effectively reduced primary tumors and the metastatic burden, similar to ICI combination therapy, through dynamic remodeling of the TIME. Furthermore, hepatitis was significantly decreased in the JX-594 and PD-1 inhibitor combination group, suggesting the potential benefit of that combination for reducing ICI-induced toxicity.

## Background

Although the prognosis of metastatic renal cell carcinoma (mRCC) is poor, the advent of targeted therapies and immuno-oncology agents has revolutionized cancer therapy and significantly improved the oncological outcomes of mRCC [1, 2]. However, only about one third of patients respond to immune checkpoint inhibitor (ICI) treatment due to the intrinsic resistance of a non-inflamed “cold” tumor immune microenvironment (TIME) to ICI treatment [3]. Research has therefore focused on changing the TIME to express immune-stimulating and tumor-suppressive phenotypes [4].

Oncolytic virus (OV) immunotherapy, a promising treatment strategy, could remodel the TIME toward a T cell–inflamed phenotype through selective infection and replication in cancer cells, as well as the induction of immunogenic cancer cell death, which eventually induces both oncolysis and systemic immune activation [5]. We previously reported the therapeutic efficacy of a systemic injection of oncolytic vaccinia virus, JX-594 (pexastimogene devacirepvec, Pexa-vec), in a murine mRCC model [6, 7].

Two immune checkpoints, cytotoxic T-lymphocyte-associated antigen 4 (CTLA-4) and programmed cell death protein-1/ligand-1 (PD-1/PD-L1), are important regulators of immune regulation and tolerance [8]. ICIs work by blocking those immune checkpoint pathways to reactivate T cell–mediated antitumor immunity [9]. ICIs have been reported to cause immune-related adverse events (irAEs), autoimmune-like disorders that occur through reactivation of cellular immunity [9, 10]. Given the increasing use of ICIs, understanding their toxicologic profile is crucial because some irAEs can be life-threatening and require rapid intervention [11].

Hepatitis is the most severe of the irAEs [12]. It is more common when CTLA-4 and PD-1/PD-L1 inhibitors are used in combination than when only PD-1/PD-L1 inhibition is used [8], which suggests that the hepatotoxic effect of CTLA-4 inhibition is higher than that of PD-1/PD-L1 inhibition.

Because OVs do not require baseline intratumoral T cell infiltration before treatment, JX-594 can be applied in combination with a PD-1/PD-L1 inhibitor [7, 13–15].

Therefore, we used a murine model to evaluate both the therapeutic and safety potential of JX-594 in combination with a PD-1 inhibitor and compared it with the combination of PD-1 and CTLA-4 inhibitors conventionally used as the first-line treatment option for mRCC. We also demonstrated that JX-594 in combination with a PD-1 inhibitor dynamically remodeled the TIME, and we investigated the combination’s immunotherapeutic potential.

## Methods

### Cell culture

The Renca murine renal cell carcinoma (RCC) cell line was purchased from the American Type Culture Collection (Manassas, VA, USA). The cells were cultured in RPMI 1640 supplemented with 10% fetal bovine serum (Gibco; Thermo Fisher Scientific, Inc., Waltham, MA, USA) and 1% penicillin/streptomycin (Sigma-Aldrich, St. Louis, MO, USA) at 37 °C in a humidified atmosphere containing 5% CO_2_.

### Tumor models and treatments

Animal experiments were conducted in accordance with the Guide to the Care and Use of Laboratory Animals approved by the Association for Assessment and Accreditation of Laboratory Animal Care and the National Institutes of Health guidelines. The experimental protocol was approved by the Institutional Animal Care and Use Committee (IACUC) of Yonsei University Health System (IACUC No. 2020–0006), following guidelines specified by the Institute of Laboratory Animal Resources Commission of the Life Sciences National Research Council in the USA. Adult male BALB/c mice (Orient Bio Inc., Seongnam, GyeongGi-Do, Korea) aged 6–7 weeks were used in this study. We used the highly pulmonary metastatic orthotopic RCC mouse model developed by our team, injecting highly selective pulmonary metastatic Renca cells (1 × 10^5^ cells/100 µL) directly into the kidney [16]. This model was then divided into early- and advanced-stage models to reproduce the International mRCC Database Consortium (IMDC) risk criteria used in clinical settings [6]. Five treatment groups were assigned to each model with eight mice per group: a control group (Control), groups treated with either JX-594 or a PD-1 inhibitor (JX-594 or anti-PD1), a group treated with PD-1 and CTLA-4 inhibitors (anti-PD1 + anti-CTLA-4), and a group treated with JX-594 and a PD-1 inhibitor (JX-594 + anti-PD1). To assess hepatitis, we utilized an early-stage mRCC model to distinctly observe the impact of the ICI combination. The administration dosage of the ICI combination was higher in early-stage mRCC models than in advanced-stage mRCC models, concurrently mitigating the effects of the tumor, including paraneoplastic syndrome. Four treatment groups with eight mice per group were assigned: a negative control group that is no-tumor-implanted mice (Negative control), a control group that is tumor-implanted mice with no treatment (Control), a group treated with PD-1 and CTLA-4 inhibitors (anti-PD1 + anti-CTLA-4), and a group treated with JX-594 and a PD-1 inhibitor (JX-594 + anti-PD1). In order to minimize potential confounders, random allocation of the mice to each group (Jee Soo Park (JSP)) and blinding (allocation: JSP, conduct of the experiment: Myung Eun Lee (MEL), outcome assessment: Keunhee Oh (KO) and Namhee Lee (NL), data analysis: JSP) was performed. The JX-594 (1 × 10^7^ plaque-forming units, SillaJen Inc., Yongin-si, Gyeonggi-do, Korea) was intraperitoneally injected every 3 days for three times. The PD-1 inhibitor (10 mg/kg, clone J43, Bio X Cell) with or without a CTLA-4 inhibitor (4 mg/kg, clone 9D9, Bio X Cell) was administered every 3 days according to the dosing schedule (7 and 4 times for early- and advanced-stage models, respectively). The dosage of PD-1 and CTLA-4 inhibitor was escalated from the recommended dosage of mRCC treatment (PD-1 inhibitor (3 mg/kg) and CTLA-4 inhibitor (1 mg/kg)) due to the intrinsic resistance to immune checkpoint blockade of Renca mouse model [7]. On the 21st day (3 weeks) after cell inoculation, the mice were sacrificed, and their kidney tissues were harvested and weighed. Their lungs were inflated with India ink to visualize lung tumor nodules.

### Immunofluorescence staining

Immunofluorescence staining was performed in both the early- and advanced-stage mRCC models on the 21st day after cell inoculation. Tissues were fixed in 10% formalin overnight and then transferred to 70% ethanol. The samples were paraffin embedded, sectioned, and stained with the following primary antibodies: rabbit anti-cluster of differentiation (CD)31 (EPR17260-263, Abcam, Cambridge, MA, USA), rat anti-CD8 (YTS169.4, Abcam), rabbit anti-PD-L1 (EPR20529, Abcam), mouse anti-cytokeratin (C11, Santa Cruz Biotechnology, Santa Cruz, CA, USA), rat anti-Gr-1 (RB6-8C5, Cell Signaling, Danvers, MA, USA), rabbit anti-CD11b (E6E1M, Cell Signaling), rat anti-F4/80 (BM8.1, Cell Signaling), rabbit anti-iNOS (D6B6S, Cell Signaling), rabbit anti-CD206 (E6T5J, Cell Signaling), rabbit anti-FOXP3 (D6O8R, Cell Signaling), or rat anti-CD4 (RM4-5, BD Pharmingen, Franklin Lakes, NJ, USA). After being washed, the slides were incubated with the following secondary antibodies: FITC-conjugated or Texas Red-conjugated anti-rabbit IgG (Vector Laboratories, Burlingame, CA, USA), FITC-conjugated anti-rat IgG (Jackson ImmunoResearch, West Grove, PA, USA), or Texas Red-conjugated anti-mouse IgG (Vector Laboratories). Then, the samples were mounted with VECTASHIELD® mounting medium (Vector Laboratories). The immunofluorescence images were captured using a Zeiss LSM700 confocal microscope (Carl Zeiss Microscopy GmbH, Jena, Germany). Staining was quantified using ImageJ software. Signal intensity was calculated as the number of positively stained pixels relative to the total number of pixels per tumor section (% positive).

### Aspartate aminotransferase (AST) and Alanine Aminotransferase (ALT) measurements

Liver enzyme activity was measured by collecting blood at baseline and then every 6 days during the course of the exposures. Serum was obtained from the blood by centrifuging at 2,500 rpm for 10 min. The serum activity of AST and ALT, biomarkers of liver injury, was measured using a Fuji Dri-Chem system (Fujifilm, Tokyo, Japan).

### Histopathological examination

Liver tissue specimens from the different groups were fixed in 10% formaldehyde for 24 h and then dehydrated and embedded in paraffin. 4-µm thick sections were cut from each paraffin-embedded tissue and stained with hematoxylin and eosin (H&E). The slides were evaluated under a light microscope (Olympus BX53) by a pathologist (Minsun Jung).

### Statistical analysis

Statistical analyses were performed using GraphPad Prism version 8.0 (GraphPad Software, Inc., La Jolla, CA, USA) and SPSS version 23.0 (IBM Corp., Armonk, NY, USA). All results are expressed as the mean ± standard deviation unless otherwise indicated. Student’s t-test was used unless the dataset did not follow a normal distribution in a Shapiro–Wilk normality test. If the dataset did not follow a normal distribution, the Mann-Whitney U test was used. All statistical tests were two-tailed, and p-values < 0.05 were considered significant.

## Results

### The therapeutic efficacy of JX-594 and ICIs in early- and advanced-stage mRCC models

**Fig. 1:**
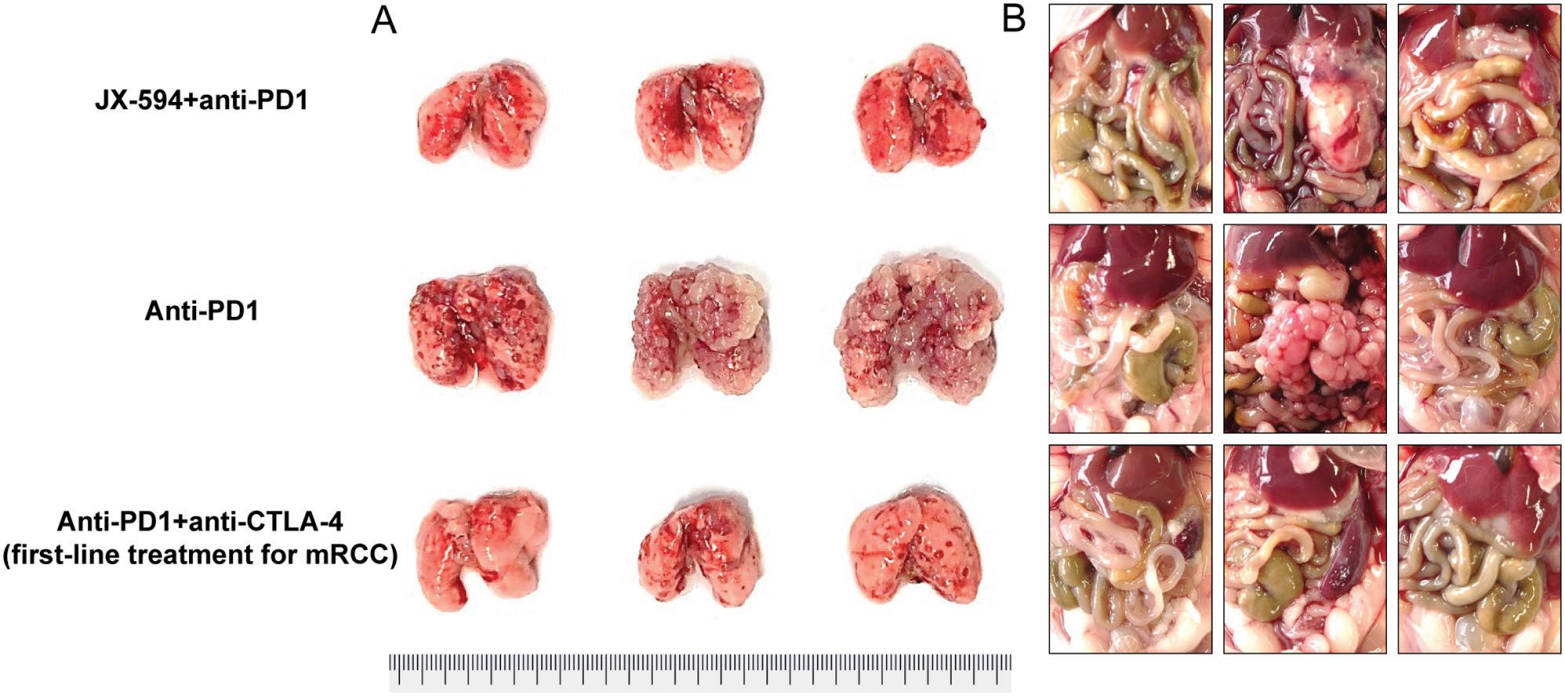
Representative images and comparisons of the tumor burden in the (**A**) lung and (**B**) parietal peritoneum of early-stage mRCC model mice treated with the JX-594 and PD-1 inhibitor combination, a PD-1 inhibitor only, or the PD-1 and CTLA-4 inhibitor combination

The combination of JX-594 and a PD-1 inhibitor worked about as well in metastatic sites such as lung metastases and peritoneal carcinomatosis as the ICI combination (Fig. 1). The group with PD-1 inhibitor monotherapy demonstrated multiple peritoneal seeding metastases on the surface of the peritoneum, accompanied by the accumulation of malignant ascites and malformed neovessels near the tumor mass (Fig. 1). However, both the combination of JX-594 and a PD-1 and ICI combination demonstrated a significantly lower number of seeding metastases, reduced neovessels, and a decreased amount of malignant ascites (Fig. 1).

**Fig. 2:**
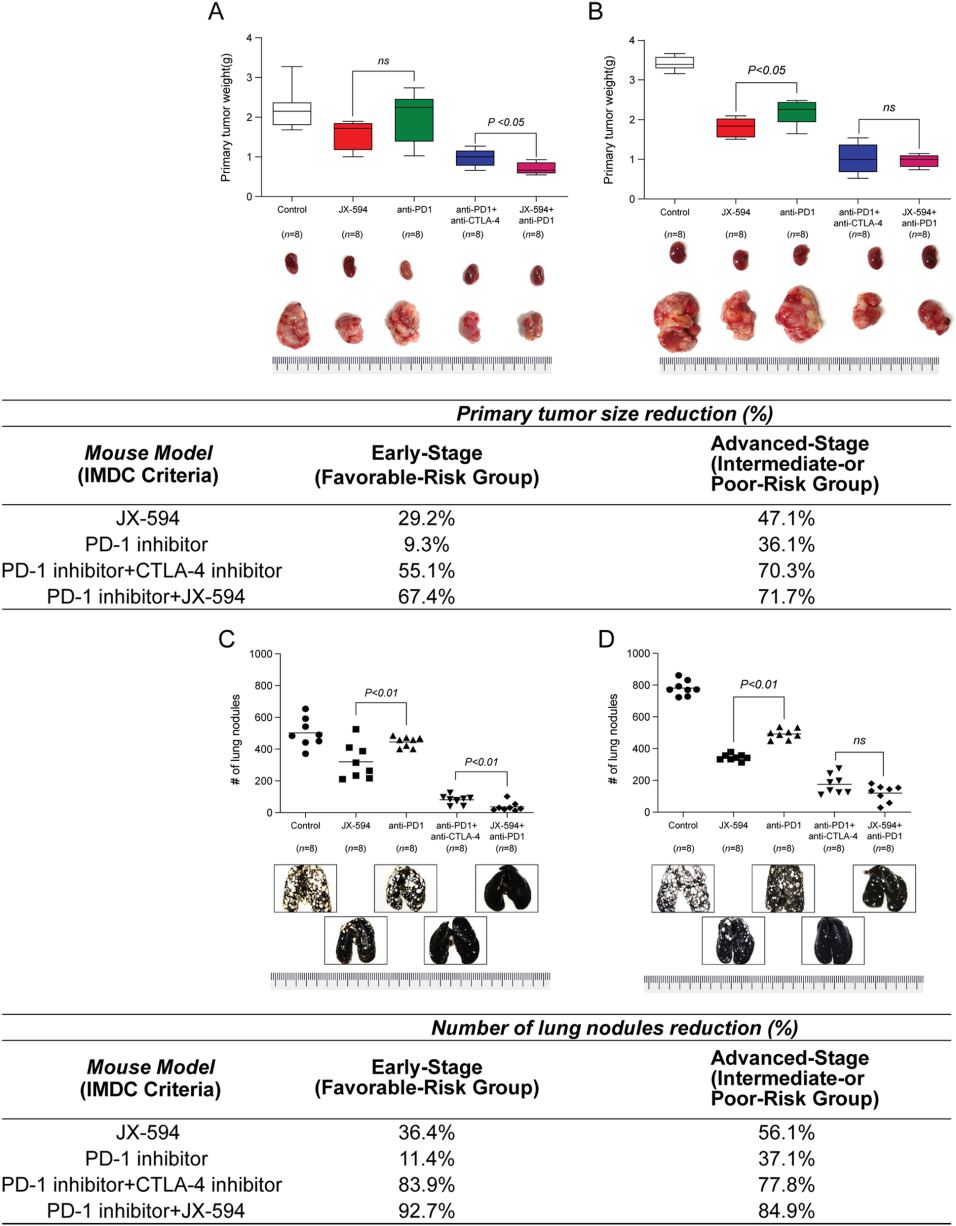
Pulmonary metastatic orthotopic renal cell carcinoma early-stage (**A** and **C**) and advanced-stage (**B** and **D**) mRCC mouse models to compare the (**A** and **B**) primary tumor weight and (**C** and **D**) number of lung nodules in the control (vehicle-treated), JX-594, PD-1 inhibitor, PD-1 and CTLA-4 inhibitor combination, and JX-594 and PD-1 inhibitor combination conditions

When we measured the primary tumor volume and number of lung nodules, the JX-594 and PD-1 inhibitor combination showed the best therapeutic efficacy in both the early- and advanced-stage mice (Fig. 2). PD-1 inhibitor monotherapy reduced the primary tumor size to some extent, but its efficacy was significantly lower than that of the other treatment modalities. In terms of lung metastases, on the other hand, PD-1 inhibitor monotherapy demonstrated fair efficacy.

The PD-1 inhibitor and JX-594 combination (mean primary tumor weight = 0.71 g) and ICI combination (mean primary tumor weight = 0.98 g) had significantly different effects on the primary tumor burden in the early-stage (*P* < 0.05) (Fig. 2A). However, JX-594 monotherapy (mean primary tumor weight = 1.55 g) and PD-1 inhibitor monotherapy (mean primary tumor weight = 1.99 g) did not differ significantly from each other (*P* = 0.11) (Fig. 2A). The overall effects of JX-594 were greater on lung metastases than on the primary tumor burden: JX-594 monotherapy worked significantly better than PD-1 inhibitor monotherapy on lung metastases (*P* < 0.01), and the JX-594 and PD-1 inhibitor combination worked better than the ICI combination (*P* < 0.01) (Fig. 2C).

In the advanced-stage model, the pronounced effects of the JX-594 and PD-1 inhibitor combination were reduced compared with the ICI combination, with no significant differences between the groups in primary tumor reduction or the number of lung nodules (Fig. 2B and 2D).

### Combination of JX-594 and PD-1 inhibitor elicits an enhanced anticancer effect by remodeling the TIME in early-stage mRCC

**Fig. 3:**
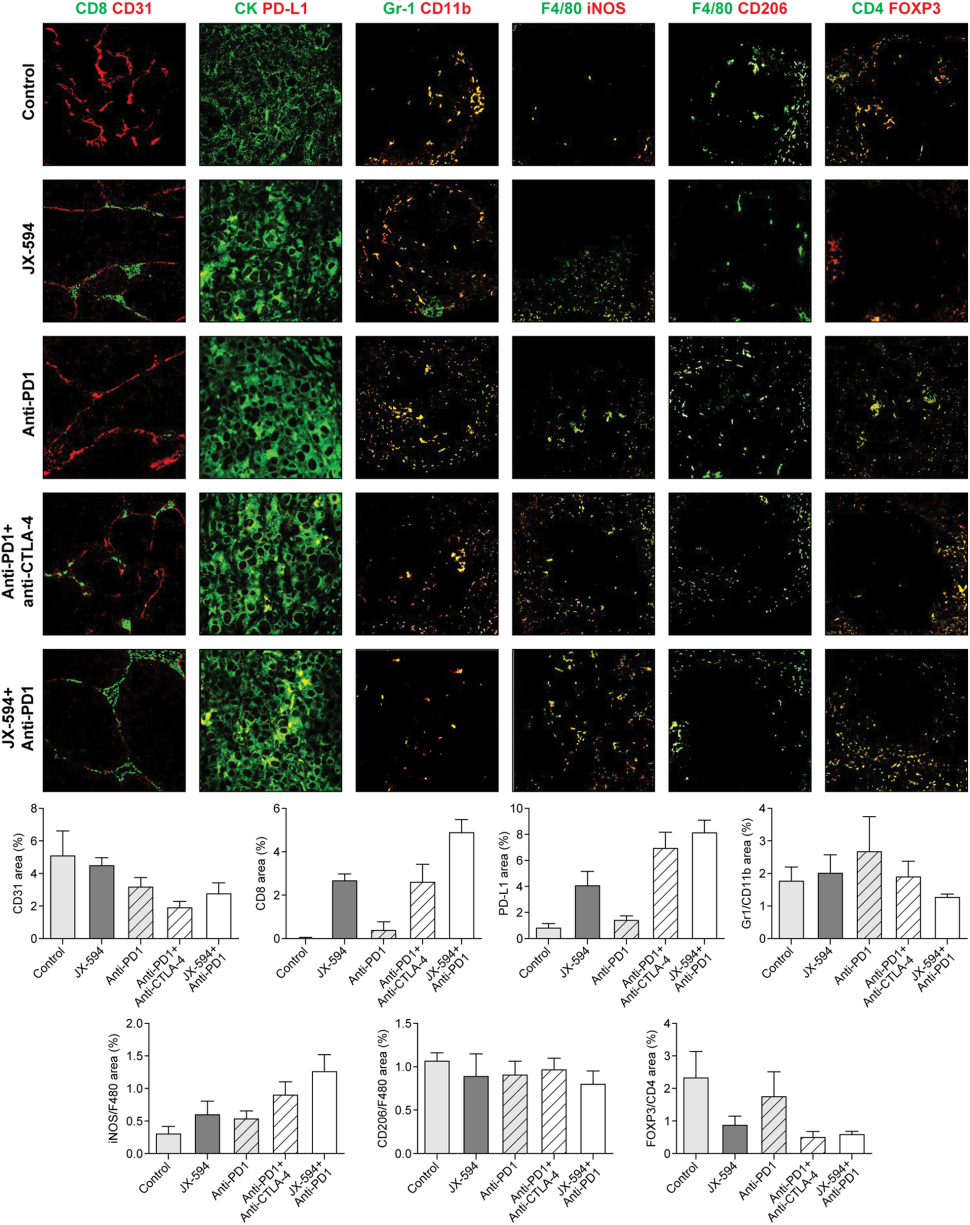
Representative images of the tumor immune microenvironment in lung metastatic sites of early-stage mRCC model mice treated with vehicle (control), JX-594, PD-1 inhibitor, PD-1 and CTLA-4 inhibitor combination, and JX-594 and PD-1 inhibitor combination. Tumor sections were stained for CD8^+^ cytotoxic T cells, CD31^+^ blood vessel, PD-L1^+^CK^+^ immune checkpoints in tumor cells, Gr1^+^CD11b^+^ myeloid cells, F4/80^+^iNOS^+^ M1 tumor-associated macrophages (TAMs), F4/80^+^CD206^+^ M2 TAMs, CD4^+^FOXP3^+^ regulatory T cells

Given the limited therapeutic activity of ICI treatment in certain mRCC patients with a non-inflamed TIME [3], we have investiagetd the effect of combining of JX-594 with a PD-1 inhibitor in TIME. The JX-594 and PD-1 inhibitor combination most significantly enhanced the recruitment of CD8 + T cells, with a 1.9-fold increase compared with the ICI combination, while concomitantly increasing PD-L1 (Fig. 3). Meanwhile, myeloid-derived suppressor cells (MDSCs) and regulatory T cells (Tregs) were most downregulated in the group treated with the JX-594 and PD-1 inhibitor combination (Fig. 3). PD-1 inhibitor monotherapy significantly increased both MDSCs and Tregs, by 2.2- and 2.9-fold, respectively, compared with the JX-594 and PD-1 inhibitor combination. The JX-594 and PD-1 inhibitor combination upregulated M1 tumor-associated macrophages (TAMs), with a 2.3-fold increase compared with PD-1 inhibitor monotherapy, and downregulated M2 TAMs (Fig. 3).

### Similar anticancer effects between the JX-594 and PD-1 inhibitor combination and ICI combination via TIME remodeling in advanced-stage mRCC model

**Fig. 4:**
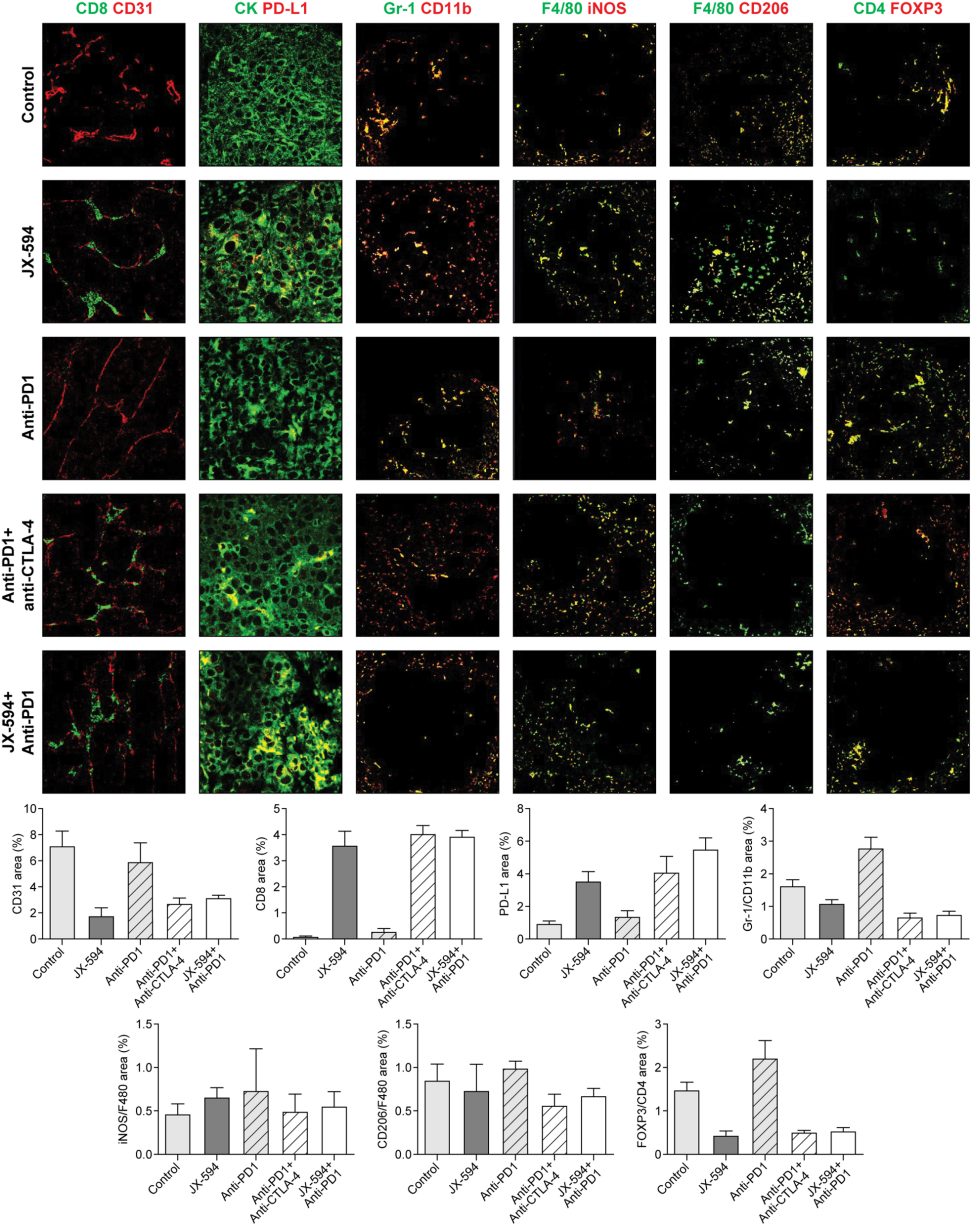
Representative images of the tumor immune microenvironment at lung metastatic sites in advanced-stage mRCC model mice treated with vehicle (control), JX-594, PD-1 inhibitor, PD-1 and CTLA-4 inhibitor combination, and JX-594 and PD-1 inhibitor combination. Tumor sections were stained for CD8^+^ cytotoxic T cells, CD31^+^ blood vessel, PD-L1^+^CK^+^ immune checkpoints in tumor cells, Gr1^+^CD11b^+^ myeloid cells, F4/80^+^iNOS^+^ M1 tumor-associated macrophages (TAMs), F4/80^+^CD206^+^ M2 TAMs, CD4^+^FOXP3^+^ regulatory T cells

CD8 + T cells were highly infiltrated in the groups treated with JX-594 monotherapy, the ICI combination, and the JX-594 and PD-1 inhibitor combination (Fig. 4), without significant differences. The trend of TIME changes in PD-L1, MDSC, M1 and M2 TAMs, and Tregs caused by the ICI combination and JX-594 and PD-1 inhibitor combination were similar (Fig. 4).

### Treatment with the ICI combination caused significant liver injury, but the JX-594 and PD-1 inhibitor combination caused only mild liver injury

**Fig. 5:**
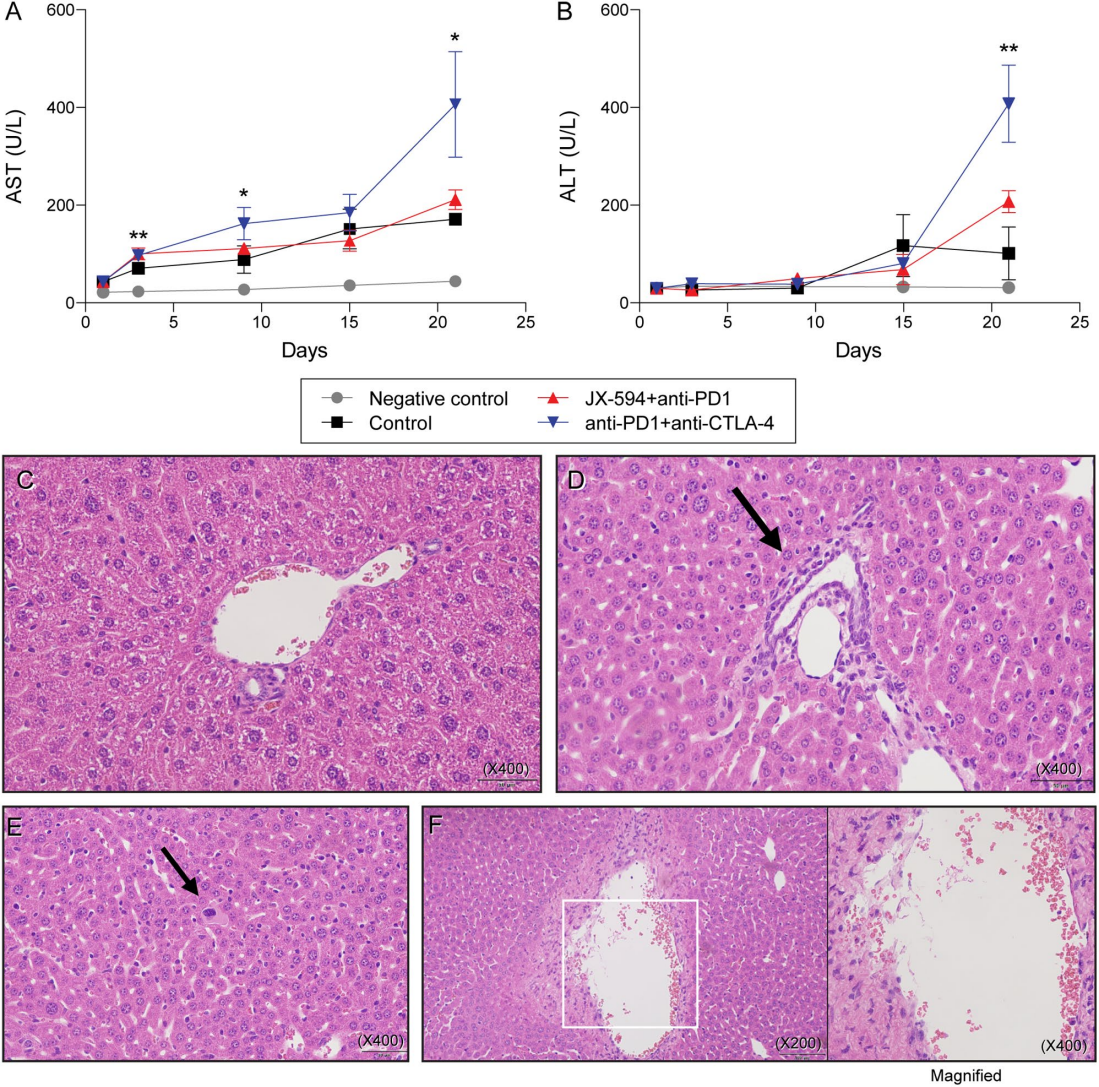
Liver injury, as measured by (**A** and **B**) AST and ALT levels in serum, and (**C**, **D**, **E**, and **F**) histopathological examination in early-stage mRCC model. (**A**) AST levels in mice from the different groups (negative control group; control group; JX-594 and PD-1 inhibitor combination group; PD-1 and CTLA-4 inhibitor combination group). (**B**) ALT levels in mice from the different groups (negative control group; control group; JX-594 and PD-1 inhibitor combination group; PD-1 and CTLA-4 inhibitor combination group). The negative control group contained mice that did not receive a cancer cell injection. The control group contained mice that received cancer cell injection but did not receive treatment. The values represent the mean ± SE. Analyzed for statistical significance by one-way ANOVA. A *P* of < 0.05 was considered significant (*P < 0.05; **P < 0.01). Results from post-hoc analysis are described in the manuscript. Representative H&E-stained liver sections from mice treated with (**C**) the JX-594 and PD-1 inhibitor combination or (**D**, **E**, and **F**) the ICI combination. Normal portal tract (**C**), infiltration of some lymphocytes and eosinophils in the portal tract (**D**), an abnormal hepatocyte with a large degenerative nucleus, amphophilic cytoplasm, and low nucleus/cytoplasmic ratio, suggesting early necrotic process (**E**), and infiltration of some lymphocytes and eosinophils in the wall of central vein. (**F**). Black arrows indicate portal inflammation (**D**) and abnormal hepatocyte (**E**). Original magnification x200, right panel of (**F**) is magnified image (x400) of the are boxed in the left panel of (**F**). H&E: hematoxylin and eosin

The ICI combination produced significantly increased levels of AST and ALT, compared with the other groups, especially JX-594 and PD-1 inhibitor combination at day 21 (*P* < 0.05 for both AST and ALT levels) (Fig. 5A and 5B). Although the JX-594 and PD-1 inhibitor combination increased the AST and ALT levels compared with the no-tumor-implanted mice at day 21 (*P* < 0.01 for both AST and ALT levels) (Fig. 5A and 5B), the AST levels did not significantly differ from those in the tumor-implanted mice that received no treatment at day 21 (*P* = 0.06) (Fig. 5A).

The ICI combination treatment produced liver injuries of portal inflammation, necrotic hepatocytes, and central venulitis (Fig. 5D, 5E and 5F), whereas the JX-594 and PD-1 inhibitor combination group showed limited hepatic injury and mostly normal portal tracts (Fig. 5C).

## Discussion

Our study has demonstrated that the JX-594 and PD-1 inhibitor combination effectively reduced primary tumors and metastatic burdens, similar to the ICI combination. Furthermore, hepatitis, an important irAE, was significantly decreased in the JX-594 and PD-1 inhibitor combination group, suggesting that the JX-594 and PD-1 inhibitor has potential benefits for reducing ICI-induced toxicity, especially that caused by the CTLA-4 inhibitor.

We chose JX-594 as a partner for the PD-1 inhibitor because JX-594 turned the cold TIME into hot TIME by enhancing the recruitment of CD8 + T cells and increasing PD-L1 expression, as demonstrated in previous studies and our results [6, 7]. That phenomenon hinders the anticancer effects of cytotoxic T cells. Therefore, we incorporated a PD-1 inhibitor, which is ineffective in non-inflamed, T cell–insufficient tumors [7]. Combining those two modalities allows them to compensate for their respective weaknesses and thereby maximize their therapeutic effects. As expected, the JX-594 and PD-1 inhibitor combination demonstrated better therapeutic efficacy in both the primary tumor and lung metastatic sites than the ICI combination currently used as first-line treatment for mRCC.

JX-594 is a vaccinia virus derived from the Wyeth strain, a typical smallpox vaccine strain [17]. By genetically modifying this strain through deactivating its thymidine kinase (TK) gene and at the same time by inserting genes encoding human granulocyte-macrophage colony-stimulating factor (GM-CSF) at TK locus, virus selectively replicates within tumor tissue [17]. Additionally, JX-594 can activate dendritic cells (DCs) via GM-CSF expression, resulting in marked antitumor efficacies through oncolytic and immune-stimulating activities [7, 18]. When administered, JX-594 commonly induces transient Common Terminology Criteria for Adverse Events (CTCAE) Grade 1 to 3 flu-like symptoms, such as mild fever, given its viral nature [19, 20]. Overall, JX-594 therapy in clinical trials has been well-tolerated to date [19, 20].

The PD-1 inhibitor we used, nivolumab (nivo), was initially approved for combination usage with ipilimumab (ipi) in treating naïve patients with IMDC intermediate and poor risk mRCC [21]. Because little information about the efficacy and toxicity of nivo monotherapy was available, a phase II study (HCRN GU16-260-Cohort A) was performed [21]. Those authors concluded that although its efficacy appears to be less than that of combination nivo/ipi in intermediate- and poor-risk patients, favorable-risk patients had a notably high objective response rate and duration of response [21]. Furthermore, they analyzed treatment-free survival (TFS) in the same cohort because ICI treatments are associated with prolonged disease control after discontinuation, without the need for further anticancer therapy [22]. Nivolumab monotherapy, compared with salvage nivo/ipi, resulted in substantial TFS and toxicity-free TFS, especially in favorable-risk patients, further supporting the use of a PD-1 inhibitor-only regimen in this population [22]. Our study also shows the potential disease control offered by a PD-1 inhibitor in mRCC: PD-1 inhibitor monotherapy successfully controlled the disease burden in metastatic sites. However, the PD-1 inhibitor fell short of modulating the TIME into a tumor-suppressing environment.

We previously demonstrated in several studies that JX-594 can dramatically convert the TIME from a cold to a hot state by inducing Th1 responses, recruiting T cells, upregulating PD-L1, and inducing M1 polarization of myeloid cells [6, 7, 23]. Moreover, the activity levels of OVs are higher in cold tumors than hot ones because few immune cells are present to eliminate the virus, whereas hot tumors have abundant resident TILs to induce premature viral clearance [24]. Therefore, we anticipated that JX-594 would be an optimal treatment partner for a PD-1 inhibitor. Our results show that combining JX-594 with a PD-1 inhibitor formed a tumor-suppressing TIME that increased the number of tumor-infiltrating CD8 + T cells, induced polarization from tumor-promoting M2 TAMs to tumor-suppressing M1 TAMs, and decreased tumor-promoting MDSCs. Putting previous findings together with the results of this study, JX-594 emerges as an optimal treatment partner for a PD-1 inhibitor.

Clinical trials combining JX-594 and a PD-1 inhibitor are currently ongoing, and theirs results have not been disclosed yet. However, according to the interim reports, 5.7% of adverse events (AEs) were reported as CTCAE Grade 3, including fever, flu-like symptoms, blood pressure changes post-infusion, and pneumonia, most of which were transient [25]. Since these AEs are also expected with JX-594 alone, we can conclude that the combination of JX-594 and PD-1 inhibitors does not particularly increase AEs compared to JX-594 alone.

However, despite previous studies showing no increase in the incidence or severity of AEs when combining OV and ICI compared to OV or ICI monotherapy, the mechanisms of OVs and ICIs should be considered. OVs stimulate anti-tumor immune responses, and ICIs function to remove inhibitory signals on effector immune cells. Therefore, it is possible that hematological toxicities, such as neutropenia, anemia, and thrombocytopenia, may occur with combined therapy [26, 27]. Furthermore, innate and adaptive immune responses against the virus itself limits the potential of OVs [28]. Neutralizing antiviral antibodies not only prevent effective infection, but also hinder repetitive systemic administration, compromising their sustained use [28]. This is the major drawback of combining OVs with other cancer therapies [29]. Thus, SillaJen has announced the development of an improved version of OVs based on JX-594, which can evade neutralization by virus-specific antibodies [15]. Future studies are required to evaluate if this novel oncolytic vaccinia virus can overcome this issue.

In terms of hepatitis, the liver has a high capacity for immune tolerance due to its constant exposure to foreign antigens, which means that a blockade of the immune checkpoints can result in aberrant immune activation in the liver in up to 20% of patients [8]. In a meta-analysis of patients who received a PD-1/PD-L1 inhibitor, 3.39% of patients showed an increase in their AST levels (grade ≥ 3 in 0.75%), and 3.14% of patients showed an increase in their ALT levels (grade ≥ 3 in 0.70%) [11, 30]. In contrast, adding a CTLA-4 inhibitor to a PD-1 inhibitor resulted in a markedly higher incidence and severity of hepatitis: elevated AST levels in 16.7% of patients (grade ≥ 3 in 5.9%) and elevated ALT levels in 18.2% (grade ≥ 3 in 8.4%) [31]. That is consistent with our findings that the AST and ALT levels were most significantly increased in the PD-1 and CTLA-4 inhibitor combination group. CTLA-4 primarily affects T cell priming by antigen-presenting cells in lymphatic organs, whereas PD-1/PD-L1 affects T cell exhaustion on the periphery, and the difference in action sites causes the difference in the incidence and severity of hepatitis [11]. Therefore, the use of CTLA-4 requires caution, especially in those already at risk of hepatitis.

This study has several limitations. Although our study has its strength in evaluating therapeutic efficacy in models of two different stages, exact matching between the early- and advanced-stage models and the favorable and intermediate- to poor-risk IMDC criteria was not possible. Furthermore, because the IMDC risk criteria were developed in the TKI era [32], they are outdated; new criteria are needed in the immune-oncology era. Second, we could not evaluate other irAEs, such as colitis and pneumonitis, due to the scope of this study. Future studies should incorporate other irAEs with different incidences and severities.

## Conclusions

The JX-594 and PD-1 inhibitor combination effectively reduced primary tumors and metastatic burdens, similar to ICI combination therapy, through dynamic remodeling of the TIME to a tumor-suppressing environment. Furthermore, liver injury was significantly decreased in the group treated with the JX-594 and PD-1 inhibitor combination, compared with the ICI combination group, suggesting the potential benefit of combining JX-594 and a PD-1 inhibitor to reduce ICI-induced toxicity, especially that caused by CTLA-4 inhibition.

## Data Availability

No datasets were generated or analysed during the current study.

## Abbreviations

AE: Adverse Events
ALT: Alanine Aminotransferase
AST: Aspartate Aminotransferase
CD: Cluster of Differentiation (refers to cell surface markers)
CTCAE: Common Terminology Criteria for Adverse Events
CTLA-4: Cytotoxic T-Lymphocyte-Associated Antigen 4
H&E: Hematoxylin and Eosin (histological staining technique)
IACUC: Institutional Animal Care and Use Committee
ICI: Immune Checkpoint Inhibitor
IMDC: International Metastatic Renal Cell Carcinoma Database Consortium
irAEs: Immune-Related Adverse Events
JX-594: Oncolytic Vaccinia Virus (a specific treatment agent)
mRCC: Metastatic Renal Cell Carcinoma
MDSCs: Myeloid-Derived Suppressor Cells
NRF: National Research Foundation of Korea
OV: Oncolytic Virus
PD-1: Programmed Cell Death Protein-1
PD-L1: Programmed Cell Death Ligand-1
RCC: Renal Cell Carcinoma
SE: Standard Error
TFS: Treatment-Free Survival
TIME: Tumor Immune Microenvironment
Tregs: Regulatory T Cells
